# Sexual dimorphism in the serum metabolome following acute exhaustive exercise

**DOI:** 10.1186/s13293-025-00780-x

**Published:** 2025-11-06

**Authors:** Baile Wu, Chunxue Tang, Zhongxun Ren, Jiayu Qian, Yuxiao Deng, Zihan Fan, YanYan Zhang, Lijun Shi

**Affiliations:** 1 Department of Exercise Physiology, Beijing Sport University, 100084 Beijing, P.R. China; 2 Laboratory of Sports Stress and Adaptation of General Administration of Sport, Beijing Sport University, Beijing, China; 3 Key Laboratory of Sports and Physical Health of Ministry of Education, Beijing Sport University, Beijing, China

## Abstract

**Background:**

Sex differences in exercise metabolism have been recognized for decades, but the molecular metabolic landscape in which men and women reach standardized physiological exhaustion criteria remains unexplored.

**Objective:**

We systematically characterized serum metabolic sexual dimorphism following acute exhaustive exercise using standardized termination criteria.

**Methods:**

In a cross-sectional study (ChiCTR2400089036), forty healthy adults (20 males, 20 females; aged 22.4 ± 3.4 years, BMI 22.4 ± 2.1 kg/m^2^; *V̇*O_2peak_ 40.92 ± 5.69 ml/kg/min) underwent cardiopulmonary exercise testing via objective termination criteria, with female participants tested during the mid-luteal phase. Serum samples were collected at baseline, immediately post-exercise, and at 15 and 30 min post-exercise for comprehensive metabolomics and targeted triacylglycerol (TAG) analysis via mass spectrometry.

**Results:**

All participants achieved standardized exhaustion endpoints. Despite equivalent fat-free mass-normalized *V̇*O_2peak_, males expended 20% more energy per unit fat-free mass (*P* = 0.015). While both sexes showed similar numbers of altered metabolites (290–308), their molecular compositions differed markedly. Lipids comprised the largest fraction of sex-specific responses, with hypoxanthine, sarcosine, and lysophospholipids as key discriminators. Females showed sustained lipid downregulation while males demonstrated recoverable patterns. Notably, 37 TAGs showed sexually antagonistic regulation, and 41.7% of fitness-correlated metabolites exhibited opposite associations between sexes.

**Conclusions:**

This study reveals distinct metabolic response patterns between males and females when standardized exhaustion endpoints are reached. Key exercise-induced sex-discriminating metabolites were identified and opposing metabolic-fitness associations were observed between sexes. These findings emphasize the necessity of sex-stratified analysis in exercise metabolism research and metabolic biomarker interpretation.

**Plain english summary:**

While men and women respond differently to exercise, molecular differences at complete exhaustion have never been studied. We studied 20 men and 20 women who exercised to exhaustion using standardized criteria, then analyzed hundreds of blood molecules before, during, and after exercise. Although both sexes showed similar numbers of changed molecules, the specific types were remarkably different, with fat-related molecules showing the largest differences. We identified molecular markers that distinguished male from female responses, found certain fat storage molecules responded in opposite directions between sexes, and discovered that molecules like kynurenine and androsterone sulfate showed opposite fitness relationships in men versus women. These findings reveal that even at identical exhaustion levels, men and women use fundamentally different molecular strategies. By understanding these biological differences, we could develop personalized exercise and nutrition plans that work better for everyone’s biology, potentially improving health outcomes and athletic performance.

**Highlights:**

- 1. Males and females exhibit distinct serum metabolomic profiles when they reach standardized physiological exhaustion endpoints, with similar numbers of altered metabolites but different molecular compositions.
- 2. Lipid species composed the largest fraction of sex-differential responses, including LPLs, bile acids, and fatty acids, showing female-dominant sustained downregulation and male-dominant recoverable downregulation patterns.
- 3. Targeted analysis revealed the sexually antagonistic regulation of 37 triacylglycerol species. These species are characterized primarily by C50‒C52 carbon chain lengths and enrichment with monounsaturated fatty acids, particularly those containing palmitoleic acid.
- 4. Key metabolites showing sex-related differential characteristics, including hypoxanthine, sarcosine, and LPLs, were identified, providing molecular markers for sex differences in exercise responses.
- 5. Among the 115 metabolites correlated with *V̇*O_2peak_, 41.7% exhibited opposing correlations between sexes, with L-kynurenine and androsterone sulfate as key examples, highlighting the necessity of sex-stratified analysis and the limitations of pooled approaches.

**Supplementary Information:**

The online version contains supplementary material available at 10.1186/s13293-025-00780-x.

## Background

Sex differences in exercise metabolism are well established [1–3]. Females demonstrate enhanced fat oxidation, whereas males exhibit greater carbohydrate utilization during physical exertion. These differences arise from sex hormone profiles, body composition variations, and enzymatic activities [4–6]. These factors shape distinct metabolic strategies between sexes. Understanding sex-specific metabolic responses is essential for optimizing exercise prescriptions and developing personalized training interventions. Identical exercise protocols elicit markedly different adaptations between males and females.

Despite extensive research documenting sex differences in substrate utilization, the comprehensive molecular signatures underlying these differences remain poorly characterized. Current knowledge relies largely on indirect measurements such as respiratory exchange ratios, which provide limited insight into the complex metabolic networks driving sex-specific responses [4–6]. Most previous studies employed submaximal exercise protocols where fixed relative intensity prescriptions introduce substantial interindividual heterogeneity in physiological stress and metabolic demand [7, 8], confounding sex comparisons. Unlike submaximal exercise, where individuals experience vastly different physiological loads despite identical relative intensities, exercise to exhaustion represents an unbiased termination criterion that eliminates intensity-related confounders [9]. When both sexes reach their true physiological limits via objective criteria, they achieve equivalent relative stress regardless of absolute workload differences. This standardized approach raises a fundamental question: do males and females exhibit distinct metabolic responses even when they reach the same physiological state? No study has systematically profiled the serum metabolome following graded exercise to exhaustion via unbiased molecular approaches. This knowledge gap is significant because maximal exercise testing pushes metabolic systems to physiological limits, potentially revealing sex-specific molecular signatures that remain concealed during submaximal conditions [10, 11].

This study addresses this gap using standardized protocols ensuring equivalent physiological stress between sexes. We employ a standardized cardiopulmonary exercise test (CPET, Bruce protocol) [12] with objective termination criteria: respiratory exchange ratio (RER) ≥ 1.10, heart rate (HR) ≥ 90% predicted maximum, and rating of perceived exertion (RPE) ≥ 19 to ensure that all participants, regardless of sex, achieve true physiological exhaustion. We tested all female participants during the mid-luteal phase when sex hormone levels are elevated and stable [2, 13]. By matching participants for age, BMI, and FFM-corrected *V̇*O_2peak_ to minimize baseline variability, our study was designed to characterize the distinct metabolic signatures that emerge when males and females reach a standardized endpoint of objective physiological exhaustion.

Using untargeted metabolomics complemented by targeted lipidomics, we comprehensively profiled serum metabolic perturbations at multiple post-exercise timepoints. This approach allows us to test whether males and females employ fundamentally different metabolic strategies when faced with standardized physiological exhaustion endpoints [3, 14]. We hypothesize that under these controlled conditions, sex differences will manifest not only as variations in substrate utilization rates but also as distinct metabolic response patterns involving different metabolite classes, regulatory networks, and recovery trajectories. This research provides the first unbiased characterization of metabolic sexual dimorphism following exhaustive exercise in humans. Understanding these fundamental differences is essential for developing evidence-based, sex-specific approaches to exercise prescription, athletic training, and metabolic health interventions [2, 3].

## Method

### Human participants

**Fig. 1:**
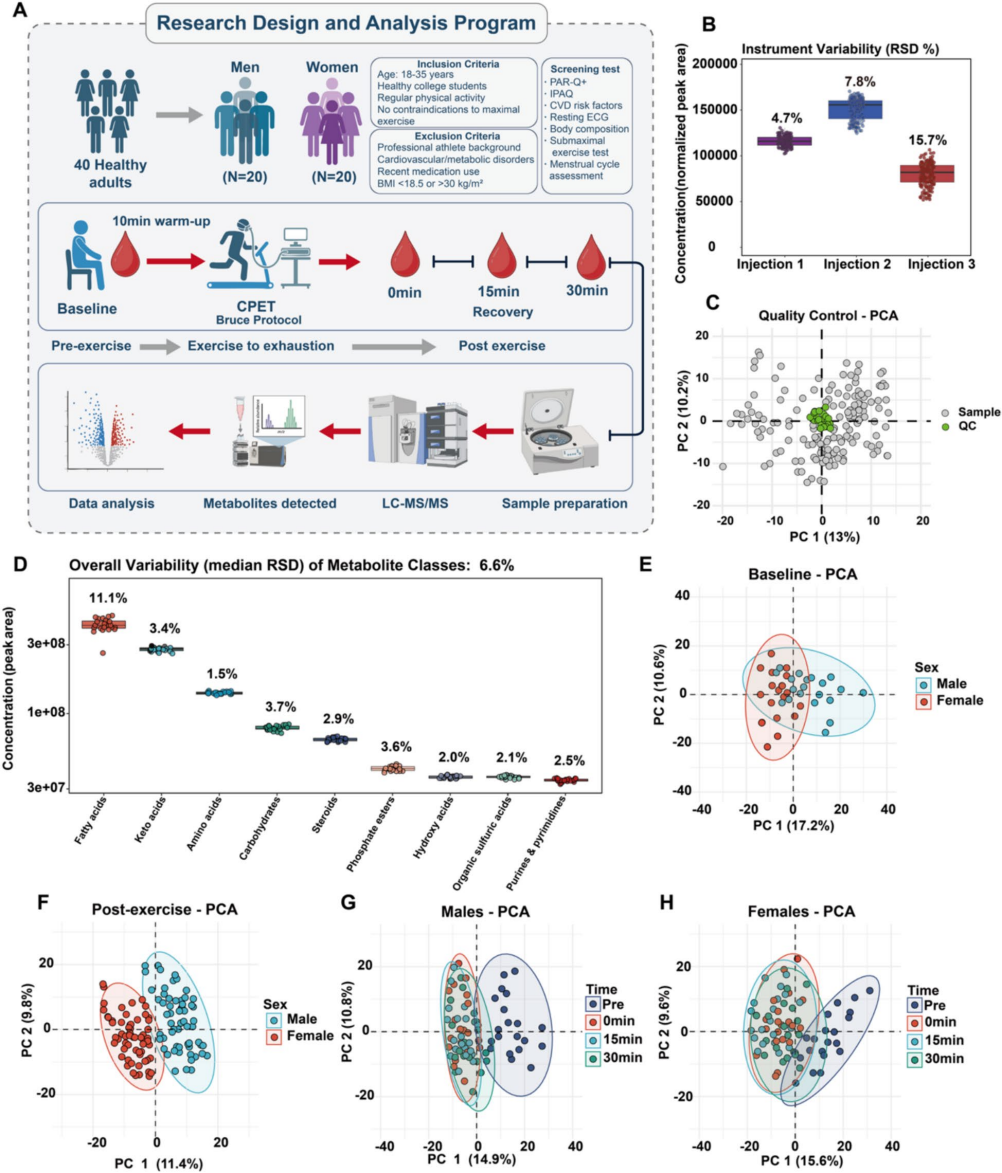
Study design, data quality control, and serum metabolome profiling. (**A**) Experimental workflow: participant screening, CPET protocol, sample collection, and metabolomics analysis. (**B**) Instrument precision: RSD of sequential injections. (**C**) PCA of individual samples and pooled QC. (**D**) Metabolite class variability: median RSD in QC samples. (**E**) PCA of baseline metabolic profiles by sex. **(F**) PCA of combined post-exercise profiles (0, 15, and 30 min) by sex. (**G-H**) PCA of temporal metabolic trajectories for males (**G**) and females (**H**)

A cross-sectional study investigated serum metabolic responses to acute exhaustive exercise between sexes. Forty healthy adults (20 men, 20 women) were recruited from Beijing Sport University and surrounding communities through campus advertisements and social media platforms were included in the final analysis **(**Fig. 1A**)**. The participant characteristics are shown in Table 1. All participants completed screening questionnaires, including the Physical Activity Readiness Questionnaire Plus (PAR-Q +), the International Physical Activity Questionnaire (IPAQ), and cardiovascular risk assessment. Body composition was assessed via dual-energy X-ray absorptiometry (DXA, GE Lunar iDXA, USA). The inclusion criteria were healthy adults aged 18–35 years; BMI 18.0–25.0 kg/m^2^; recreational activity (150–450 min/week moderate-intensity exercise per IPAQ); ability to perform maximal exercise safely; and regular menstrual cycles (25–35 days) for females. The exclusion criteria were cardiovascular disease or diabetes mellitus; metabolic, respiratory, or musculoskeletal disorders; prescription medications or supplements affecting metabolism within three months; pregnancy, breastfeeding, or irregular menstrual cycles for females; and smoking or excessive alcohol consumption (> 14 units/week). All female participants were tested during the mid-luteal phase, confirmed by a detailed menstrual history questionnaire and calendar tracking for at least two consecutive cycles prior to testing. All enrolled participants successfully completed the entire study protocol, with no withdrawals due to inability to continue throughout the experimental period.

**Table 1:** Baseline participant characteristics

	**Men** **(n = 20)**	**Women** **(n = 20)**	**Men Vs. Women** **(*P* value)**	**Effect size** **(Cohen’s |d|)**
**Anthropometric measures**				
Height (m)	1.76 ± 0.06	1.63 ± 0.05	< 0.001	2.35
Body mass (kg)	68.81 ± 8.38	58.03 ± 5.83	0.008	1.49
Age (years)	22.85 ± 3.84	21.90 ± 2.77	0.474	0.28
BMI (kg/m^2^)	22.28 ± 1.71	22.46 ± 2.4	0.862	0.09
**Body composition**				
Body fat (%)	20.14 ± 3.31	33.54 ± 3.04	< 0.001	4.22
Fat-Free Mass (kg)	54.82 ± 5.88	38.46 ± 3.01	< 0.001	3.50
WHR	0.82 ± 0.03	0.74 ± 0.04	< 0.001	2.26
**Cardiorespiratory measures**				
*V̇*O_2peak_ (ml/kg/min)	45.05 ± 4.14	36.80 ± 3.69	< 0.001	2.10
*V̇*O_2peak_ (ml/kg/FFM/min)	56.50 ± 5.65	55.45 ± 5.82	0.567	0.18
Resting HR (bpm)	73.29 ± 14.77	79.20 ± 12.20	0.248	0.44
HR_peak_ (bpm)	191.95 ± 8.79	187.30 ± 8.72	0.164	0.53
RER_Peak_	1.26 ± 0.09	1.24 ± 0.10	0.607	0.21
Endpoint RPE	19.6 ± 0.50	19.8 ± 0.41	0.176	0.44
**Exercise duration and energy cost**				
Exercise time (sec)	744.39 ± 114.04	623.30 ± 97.11	0.008	1.14
Total Energy expenditure(Kcal)	138.4 ± 36.53	80.57 ± 13.7	< 0.001	2.10
Total Energy expenditure(Kcal/kg/FFM)	2.53 ± 0.64	2.10 ± 0.38	0.015	0.82

The data are presented as the means ± SDs. Statistical comparisons between sexes were performed via independent samples t tests. BMI, body mass index; WHR, waist‒to-hip ratio; *V̇*O_2peak_, relative peak oxygen uptake; FFM, fat-free mass; HR, heart rate; RER, respiratory exchange ratio

### Experimental procedures and pretest standardization

To familiarize participants with the cardiopulmonary exercise testing (CPET) protocol, all participants received detailed instructions and equipment orientation without physical exercise practices to avoid potential metabolic interference. This included demonstration of breathing techniques, familiarization with the mouthpiece, explanation of the incremental exercise protocol, and practice with the rating of the perceived exertion scale. The participants consumed a standardized evening meal at 20:00 on the day preceding exercise testing to control for acute dietary influences. Energy requirements were calculated using the Mifflin-St Jeor equation with FFM adjustments. The standardized meal provided 55% carbohydrates, 20% protein, and 25% fat. Participants refrained from vigorous physical activity and avoided caffeine and alcohol consumption for 72 h prior to testing. On the morning of the formal test, the participants performed a single acute exhaustive exercise bout while in a fasted state. The participants were scheduled according to their enrollment numbers, with testing conducted between 7:00 and 10:00 AM to control for circadian effects. Fasting began after the standardized evening meal, ensuring an overnight fasting period of 10–12 h (water intake permitted). All testing was conducted in a climate-controlled laboratory maintained under standard conditions (temperature, 20–22 °C; relative humidity, 40–60%; standard atmospheric pressure) suitable for exercise testing. Qualified medical personnel were present throughout all testing sessions to ensure participant safety and protocol adherence.

### Cardiopulmonary exercise testing protocol

CPET was performed via the standard Bruce protocol on a motorized treadmill [12]. All participants completed a standardized 10 min warm-up guided by trained experimental personnel. The Bruce protocol commenced at 1.7 mph (2.7 km/h) with a 10% grade, with the speed and inclination incrementally increased every 3 min according to established stages until volitional exhaustion. Throughout the test, gas exchange parameters, including oxygen uptake (*V*O₂), carbon dioxide production (*V*CO₂), and the RER, were continuously monitored via a high-resolution metabolic gas analysis system (Cortex MetaLyzer® 3B, Germany). Heart rate (HR) was recorded continuously via a wireless heart rate monitor (Polar V800, Polar Electro, Finland). RPE was assessed every 2 min via the Borg 6–20 scale. The exercise termination criteria followed American College of Sports Medicine (ACSM) guidelines to ensure true maximal effort without sex bias [12]. The criteria included the following: (1) RER ≥ 1.10; (2) HR ≥ 90% of the age-predicted maximum (220-year-old); (3) RPE ≥ 19; and (4) *V̇*O_2_ plateau or decline despite an increasing workload. All participants, regardless of sex, were required to meet these standardized criteria to ensure consistent termination endpoints. The total energy expenditure was estimated from breath-by-breath gas exchange data via the Weir equation: energy expenditure (kcal/min) = 3.9 × *V*O₂ (L/min) + 1.1 × *V*CO₂ (L/min) [12]. Minute-by-minute energy expenditure was calculated throughout the graded exercise test, and the total energy cost was obtained by integrating these values from test initiation to volitional exhaustion.

### Blood collection and sample preparation

Venous blood samples (5 mL per timepoint) were collected at four timepoints: baseline (prior to warm-up procedures), immediately post-exercise (within 2 min), and at 15 and 30 min post-exercise. Blood was collected into red-top BD Vacutainer tubes without anticoagulants. After 30–60 min at room temperature, the samples were centrifuged at 3000 × g for 15 min at 4 °C. The separated serum was transferred to labeled 1.5 mL cryogenic tubes and stored at −80 °C until analysis. All the samples were processed within 2 h of collection and subjected to a single freeze‒thaw cycle.

### Metabolite extraction

Serum samples were thawed at 4 °C and vortexed briefly, followed by centrifugation to remove any precipitates. For metabolite extraction, 100 µL of serum was mixed with 400 µL of ice-cold methanol containing 0.28 mM phenylhydrazine for α-keto acid derivatization [15]. The samples were vortexed and incubated at −20 °C for 30 min and then centrifuged at 12,000 rpm for 10 min at 4 °C. The supernatant was transferred to clean 1.5 mL tubes and dried using a SpeedVac concentrator under OH mode. The dried extracts were reconstituted in 100 µL of 5% acetonitrile, vortexed thoroughly, and centrifuged at 12,000 rpm for 10 min at 4 °C to remove particulates. The clear supernatant was transferred to LC‒MS vials and analyzed immediately via ultrahigh-performance liquid chromatography (Agilent 1290 II, Agilent Technologies, Germany) coupled with high-resolution mass spectrometry (5600 Triple TOF Plus, AB Sciex, Singapore). The derivatization efficiency was confirmed via standard analytical methods to ensure consistent sample preparation across all batches.

### Lipid extraction

Lipids were extracted from 50-µL serum samples via a modified Bligh and Dyer method as previously described [16]. Briefly, 750 µL of chloroform:methanol (1:2, v/v) was added to the serum samples, which were subsequently incubated at 1500 rpm for 30 min at 4 °C. Following incubation, 350 µL of deionized water and 250 µL of chloroform were added to induce phase separation. The samples were subsequently centrifuged at 12,000 rpm for 10 min at 4 °C, after which the lower organic phase containing lipids was carefully transferred to a clean tube. Lipid extraction was repeated by adding 450 µL of chloroform to the remaining aqueous phase, followed by centrifugation under the same conditions. The lipid extracts from both extractions were pooled and dried in a SpeedVac concentrator under OH mode. The dried lipid extracts were stored at −80 °C until further analysis.

### Untargeted metabolomic analysis

Untargeted metabolomics analysis was conducted via an Agilent 1290 II UPLC coupled with a Sciex 5600 + quadrupole-TOF MS. For reversed-phase liquid chromatography (RPLC), polar metabolites were separated on a Waters ACQUITY HSS-T3 column (3.0 × 100 mm, 1.8 μm), whereas a Waters ACQUITY BEH Amide column (2.1 × 100 mm, 1.7 μm) was utilized for hydrophilic interaction liquid chromatography (HILIC). The MS parameters for detection were as follows: ESI source voltage positive ion mode, 5.5 kV; negative ion mode, − 4.5 kV; vaporizer temperature, 500 °C; drying gas (N2) pressure, 50 psi; nebulizer gas (N2) pressure, 50 psi; and curtain gas (N2) pressure, 35 psi. The scan ranges were set at m/z 60–700 during RPLC and m/z 70–800 during HILIC analysis. The information-dependent acquisition mode was used for MS/MS analyses with the collision energy set at (±) 35 ± 15 eV. Quality control (QC) samples were prepared by pooling aliquots from all samples into a mixed solution, which was injected between every ten actual samples [17, 18]. The coefficient of variation (CV) for major metabolite classes was determined by calculating the median relative standard deviation (median RSD) of the metabolites. Instrument variability was assessed by calculating the RSD of internal standard metabolites. Data acquisition and processing were performed via Analyst® TF 1.7.1 software (AB Sciex). All detected ions were extracted via MarkerView 1.3 in Excel, and the isotopic peaks were filtered. PeakView 2.2 was applied to extract MS/MS data and perform comparisons with metabolite databases (AB Sciex, HMDB) and standard references for ion annotation.

### Targeted lipidomic analysis for triacylglycerols

Targeted triacylglycerol (TAG) analysis was performed via Jasper HPLC coupled with Sciex TRIPLE QUAD 4500 MD as previously described [19]. Lipid separation was achieved via normal-phase HPLC via a TUP-HB silica column (150 × 2.1 mm, 3 µm) with mobile phase A (chloroform:methanol:ammonium hydroxide, 89.5:10:0.5) and mobile phase B (chloroform:methanol:ammonium hydroxide:water, 55:39:0.5:5.5). Multiple reaction monitoring (MRM) transitions were optimized for quantitative analysis of individual TAG species. TAG quantification was performed using deuterated internal standards, primarily TAG(16:0)3-d5 (CDN Isotopes), with additional standards selected on the basis of structural similarity to target analytes to ensure accurate quantification across the range of TAG species detected. Data acquisition and processing were performed via Analyst software (AB Sciex).

### Statistical analysis and data visualization

**Fig. 2:**
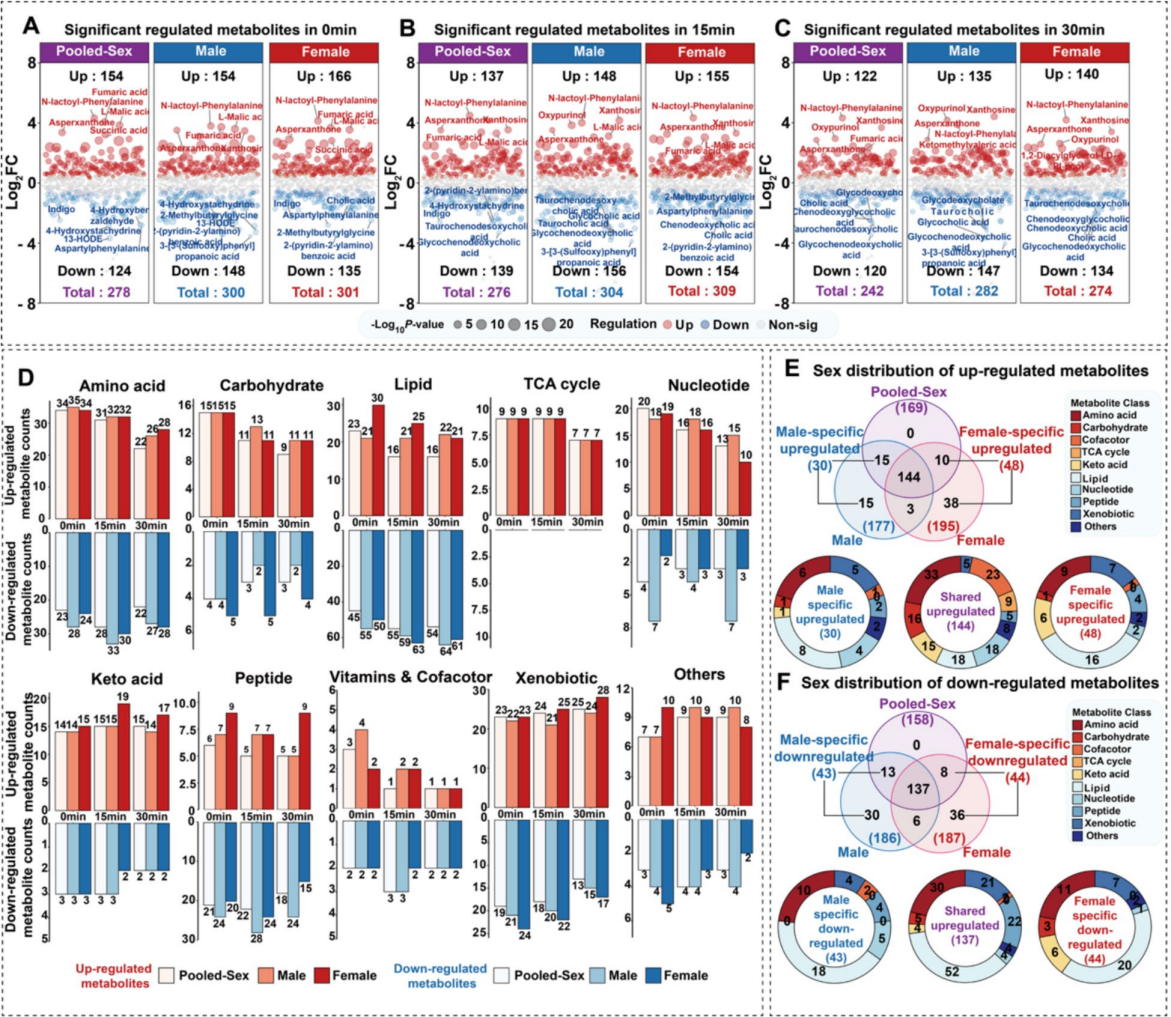
Overall characteristics and sex differences in the serum metabolome following acute exhaustive exercise. (**A-C**) Volcano plots of significantly regulated metabolites at 0, 15, and 30 min post-exercise: pooled sex, male specific, and female-specific analyses (red: upregulated; blue: downregulated). (**D**) Differentially abundant metabolite counts by chemical class and time point. (**E–F**) Sex distribution of upregulated (**E**) and downregulated (**F**) metabolites: Venn diagrams showing male specific, female specific, and shared metabolites; donut charts showing chemical class compositions

**Fig. 3:**
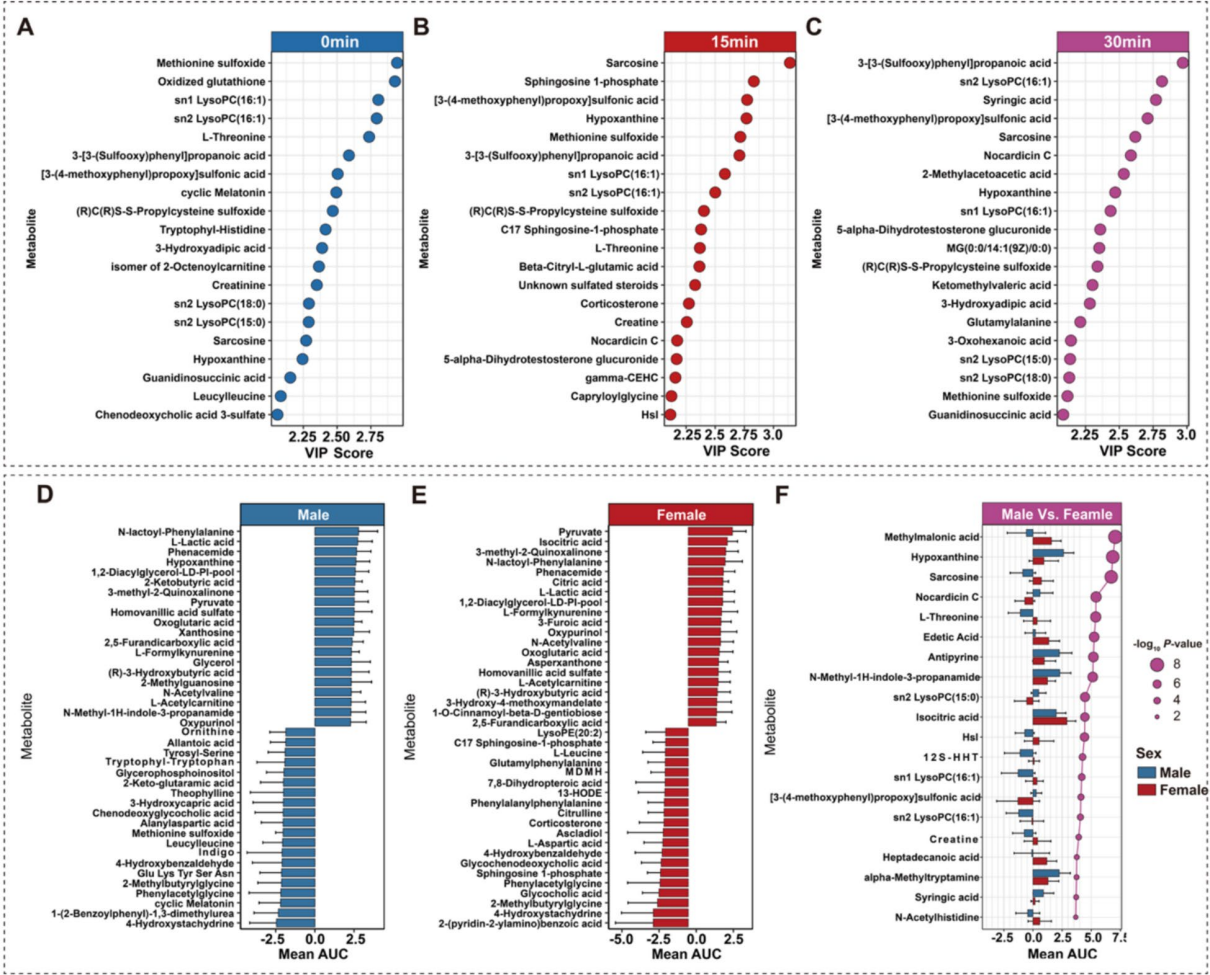
Identification of key sex-differentiated metabolites via multistrategy analysis. (**A-C**) Top 20 metabolites ranked by OPLS-DA VIP scores at 0, 15, and 30 min post-exercise. (**D-E**) Top 20 most upregulated and downregulated metabolites according to the mean AUC for males (**D**) and females (**E**). (**F**) The top 20 metabolites with the greatest sex differences in the mean AUC; the bar direction indicates a greater cumulative response, and the point size represents the -log10 adjusted *P*

**Fig. 4:**
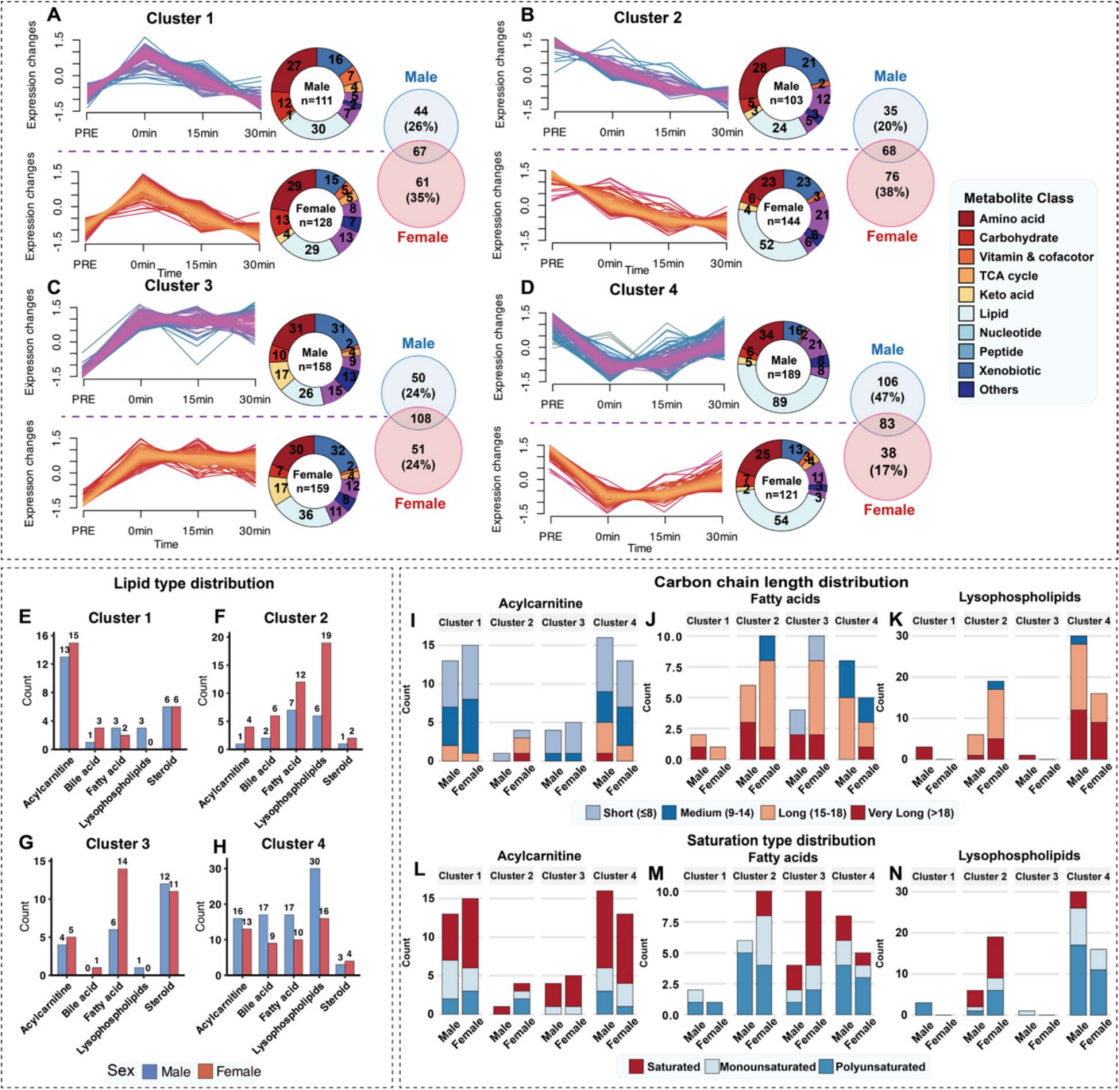
Sex-dimorphic temporal dynamics and metabolic network architecture in response to acute exercise. (**A-D**) Four major metabolic patterns from fuzzy c-means clustering, Recoverable upregulation (**A**), Sustained downregulation (**B**), Elevated and maintained (**C**), and Recoverable downregulation**(D)**, donut charts show metabolite counts and chemical class distributions by sex. (**E–H**) Sex-specific distribution of five major lipid classes within temporal clusters. (**I-K**) Carbon chain length distributions of acylcarnitines **(I)**, FAs **(J)**, and LPLs (**K**). (**L‒N)** Saturation status distributions of acylcarnitines (**L**), FAs (**M**), and LPLs (**N**)

**Fig. 5:**
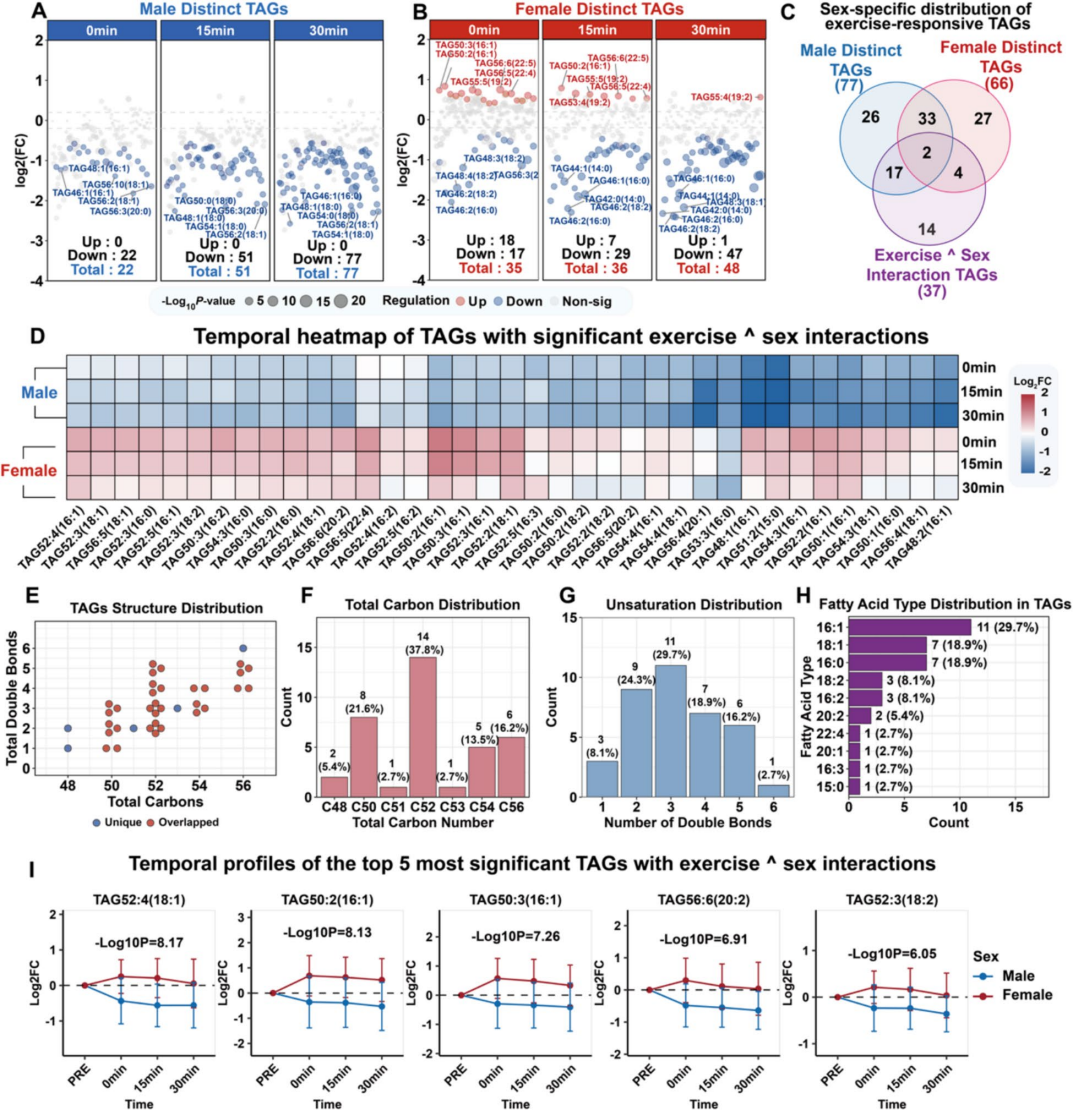
Targeted profiling of serum TAGs reveals a sexually antagonistic response to acute exercise. (**A-B**) Volcano plots of TAG abundance changes at 0, 15, and 30 min post-exercise for males and females; node color indicates the regulation direction, and node size represents the -log_10_ *P* value. **(C)** Venn diagram of exercise-responsive TAGs: male specific, female specific, and sex interaction effects. (**D**) Temporal heatmap of 37 TAGs with significant exercise-by-sex interactions; rows grouped by sex and timepoint; color intensity indicates log_2_FC magnitude. (**E–H**) Structural characterization of 37 sex-dimorphic TAGs: total carbon vs. double bonds (**E**), carbon number distribution (**F**), degree of unsaturation (**G**), and fatty acid composition (**H**). (**I**) Temporal profiles of the top five TAGs ranked by exercise-by-sex interaction adjusted *P* values; blue lines represent males, red lines represent females, and shaded areas represent standard deviations

**Fig. 6:**
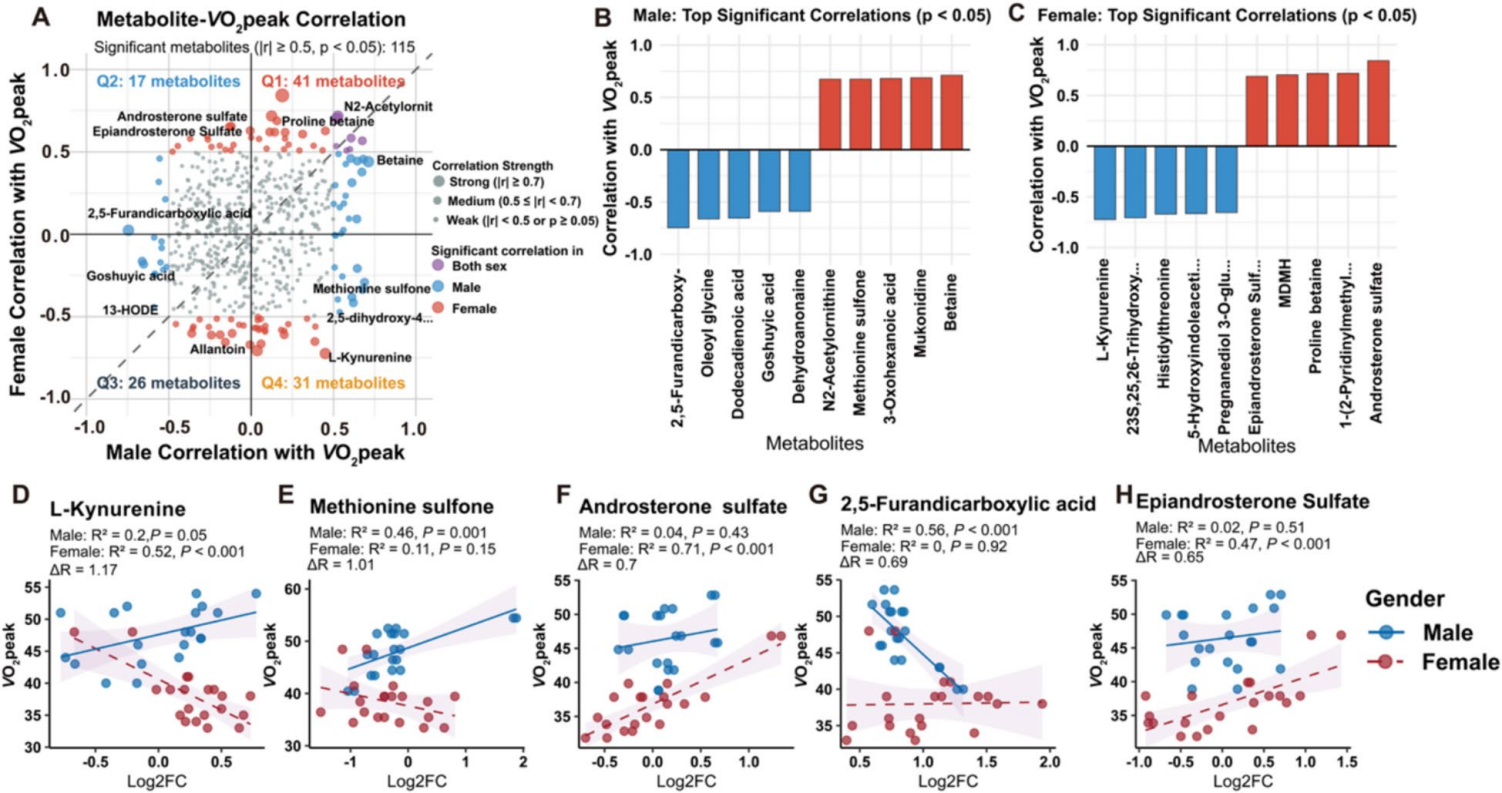
Sex-dimorphic metabolic signatures of the *V̇*O_2peak_. (**A**) Pearson correlation coefficients between maximum post-exercise metabolite perturbations and *V̇*O_2peak_ comparing males vs. females; node color indicates a significant pattern; node size represents average correlation strength |r|. (**B-C**) Most significantly correlated metabolites with the *V̇*O_2peak_ for males (**B**) and females (**C**); bars show correlation coefficients. (**D**-**H**) Correlations for the five metabolites with the greatest sex differences (ΔR): L-kynurenine (**D**), methionine sulfone (**E**), androsterone sulfate (**F**), 2,5-furandicarboxylic acid (**G**), and epandrosterone sulfate (**H**). Blue represents males, and red represents females; shaded areas represent 95% confidence intervals. The sex difference is quantified as *ΔR*, which is calculated as the absolute difference between the male and female Pearson correlation coefficients (*ΔR* = ∣*r*_*male*_ − *r*_*female*_∣)

Sample size was determined using G*Power (version 3.1.9.7) for repeated-measures ANOVA, assuming an effect size of f = 0.25, statistical power of 0.95, α = 0.05, and correlation among repeated measures of 0.5, yielding a minimum of 18 participants per group. All reported *P* values were adjusted for multiple comparisons via the Benjamini‒Hochberg method to control the false discovery rate (FDR) at α = 0.05, unless otherwise specified. Metabolomic and lipidomic data were processed via R (v4.4.1) and visualized via Adobe Illustrator 2025 (v29.01). Data underwent normalization, standardization, quality control assessment, and missing value imputation following established protocols [20]. Statistical analysis followed a hierarchical approach in which log2-fold change (log_2_FC) values were calculated for each metabolite at post-exercise timepoints (0, 15, and 30 min) relative to baseline. Independent t tests were used to compare baseline characteristics between sexes **(**Table 1**)**. Principal component analysis (PCA) was used to assess overall metabolic patterns and sex-related separation **(**Fig. 1**)**. Paired t tests were used to compare metabolite levels at each timepoint with baseline values. Metabolites with adjusted *P* < 0.05 and | log_2_FC |> 0.2 were considered significantly altered **(**Fig. 2**)**. Two-way repeated-measures ANOVA was performed on log_2_FC datasets via the "car" R package (v3.1–2) with the factors of sex, time, and their interaction **(**Fig. 3**)**. Variable importance in projection (VIP) analysis was performed on log_2_FC datasets via the "mixOmics" R package (v6.24.0), and area under the curve (AUC) analysis was used to identify sex-dependent metabolites significantly perturbed by exercise (Fig. 3) [10]. Fuzzy c-means clustering via the "Mfuzz" R package (v2.62.0) was applied to complete log_2_FC datasets with the optimal cluster number (n = 4) and fuzzification parameter m = 2.0. Metabolite correlation networks were constructed via the "igraph" R package (v1.5.1) with Pearson correlation coefficients (|*r*|> 0.7, *P* < 0.05) from pooled post-exercise log_2_FC values, which revealed metabolites with > 10 connections **(**Fig. 4**)**. The targeted TAG data were subjected to differential analysis via paired t tests and two-way repeated-measures ANOVA to identify sex-dimorphic TAG responses, with subsequent structural characterization **(**Fig. 5**)**. Correlations between the maximum absolute log_2_FC values across all post-exercise timepoints and the *V̇*O_2peak_ were assessed via Spearman correlation with Benjamini‒Hochberg correction **(**Fig. 6**)**.

## Results

### Participant characteristics and data quality assessment

Forty participants (20 males, 20 females) completed all the experimental procedures **(**Fig. 1A**)**. All the participants reached physiological exhaustion, as confirmed by objective criteria: RER_peak_ ≥ 1.24 (males: 1.26 ± 0.09; females: 1.24 ± 0.10; *P* = 0.607), HR_peak_ ≥ 187 bpm (males: 191.95 ± 8.79; females: 187.30 ± 8.72 bpm; *P* = 0.164), and endpoint RPE ≥ 19 (males: 19.6 ± 0.50; females: 19.8 ± 0.41; *P* = 0.176). Baseline anthropometric and physiological characteristics are presented in Table 1. The male and female groups were well matched for age (22.85 ± 3.84 vs. 21.90 ± 2.77 years; *P* = 0.474) and BMI (22.28 ± 1.71 vs. 22.46 ± 2.4 kg/m^2^; *P* = 0.862). Males presented a greater *V̇*O2_peak_ (45.05 ± 4.14 vs. 36.80 ± 3.69 ml/kg/min; *P* < 0.001) and a longer time to exhaustion (744.39 ± 114.04 vs. 623.30 ± 97.11 s; *P* = 0.008). Consequently, total energy expenditure was greater in males, a difference that remained significant after normalization for FFM (2.53 ± 0.64 vs. 2.10 ± 0.38 kcal/kg/FFM; *P* = 0.015). However, no significant sex-based difference was observed in the *V̇*O2_peak_ when the FFM was normalized (56.50 ± 5.65 vs. 55.45 ± 5.82 ml/kg/FFM/min; *P* = 0.567).

The analytical platform demonstrated exceptional precision and reproducibility. The sequential injection variability ranged from 4.7% to 15.7%, whereas the pooled quality control samples presented a median RSD of 6.6% across major metabolite classes **(**Fig. 1B, D**)**. PCA of pooled quality control samples demonstrated tight clustering, demonstrating stable analytical performance across the entire experimental run **(**Fig. 1C**)**. PCA of baseline serum metabolic profiles revealed sex-specific metabolic signatures, with partial separation observed along the first principal component. This separation was enhanced when all post-exercise timepoints were incorporated, demonstrating exercise-induced amplification of metabolic dimorphism. Both sexes presented distinct time-dependent metabolic changes from their preexercise baseline values. Post-exercise samples were separated along the first principal component **(**Fig. 1E-H**)**.

### Overall metabolic response characteristics and sex differences

Significantly altered metabolites were defined as those exhibiting adjusted *P* < 0.05 and |log_2_FC|> 0.2 at any post-exercise timepoint relative to baseline. Changes from baseline were analyzed at 0, 15, and 30 min post-exercise to assess temporal metabolic changes and sex-specific response patterns. Volcano plot analysis comparing sex-stratified and pooled sex analyses revealed increased metabolite detection when biological sex was incorporated as a variable **(**Fig. 2A**‒C)**. Sex-stratified analysis revealed that 274–309 significantly regulated metabolites across timepoints, representing a 13–14% increase over pooled sex analysis (242–272 metabolites). The total counts of significantly altered metabolites were comparable between sexes (males: 290–308; females: 274–309) across timepoints.

Chemical classification analysis demonstrated that both sexes exhibited upregulation of identical metabolite classes, including amino acids, lipids, and TCA cycle intermediates, whereas others (lipids and keto acids) were downregulated **(**Fig. 2D**)**. Venn diagrams were used to quantify unique and shared metabolites across post-exercise timepoints **(**Fig. 2E-F**)**. Among the upregulated genes, 30 were male-specific (16.9% of total upregulated males), 48 were female-specific (24.6% of total upregulated females), and 144 were shared **(**Fig. 2E**)**. Among the downregulated genes, 43 were male-specific (23.1%), and 44 were female-specific (23.5%), with 137 being shared **(**Fig. 2F**)**. Lipids comprised the largest fraction of sex-specific metabolites for both upregulation (26% male, 33% female) and downregulation (42% male, 45% female, Fig. 2E-F).

### Exercise-induced sex-discriminating metabolites

To identify sex-differentiated metabolites, three analytical strategies with distinct statistical advantages were employed to log_2_FC datasets, each generating a ranked list of the top 20 metabolites. Each approach captures different aspects of sex-dimorphic metabolic responses, OPLS-DA quantifies discrimination capacity at individual timepoints, AUC analysis measures cumulative perturbation magnitude across the recovery period, and two-way repeated-measures ANOVA detects sex‒time interaction effects.

OPLS-DA analysis identified the most discriminating metabolites at each timepoint, with VIP scores of 2.0–3.0 **(**Fig. 3A-C**)**. AUC analysis revealed distinct sex patterns. Among the top 20 upregulated metabolites, 14 (70%) were shared between sexes. However, downregulated metabolites showed minimal overlap, with only 4 species common to both sexes **(**Fig. 3D, E**)**. The overlapping upregulated metabolites included glycolysis products (L-lactic acid, pyruvate) [21]; the TCA cycle intermediate (oxoglutaric acid, citric acid, isocitric acid) [22]; a ketone body ((R)−3-hydroxybutyric acid) [23]; a marker of fatty acid oxidation (L-acetylcarnitine) [24]; and other exercise-induced factors validated in other datasets, such as N-lactoyl-phenylalanine [25] and N-acetylvaline [20] **(**Fig. 3D, E**)**. Further AUC analysis pinpointed metabolites with the greatest sex differences in cumulative response (-log10*P* 2.0 to 8.0, Fig. 3F). Two-way repeated-measures ANOVA identified metabolites with the strongest sex main effects (-log10*P* > 5.78, Figure S1A). Six metabolites—hypoxanthine, sarcosine, methionine sulfoxide, sn1-LysoPC(16:1), sn2-LysoPC(16:1), and 3-[3-(sulfooxy)phenyl]propanoic acid—consistently appeared in multiple ranked lists **(**Fig. 3A-C, 3F**, **Figure S1A**)**.

### Sex-dimorphic metabolic recovery patterns and networks

Fuzzy c-means clustering identified four distinct temporal response patterns with marked sex-specific distributions **(**Fig. 4A-D**).** Cluster 1 (Recoverable Upregulation) contained similar metabolite counts between sexes (males: 111, females: 128, Fig. 4A); Cluster 2 (Sustained Downregulation) was female-dominant (males: 103, females: 144, Fig. 4B); Cluster 3 (Elevated-and-Maintained) showed nearly equal counts (males: 158, females: 159, Fig. 4C); and Cluster 4 (Recoverable Downregulation) was male-dominant (males: 189, females: 121, Fig. 4D). Metabolite class analysis revealed that lipids were disproportionately distributed by sex, particularly in Cluster 2 (males: 24, females: 52) and Cluster 4 (males: 89, females: 54), whereas the TCA cycle, amino acid, and nucleotide metabolites were more evenly distributed.

Given that lipids were female-dominant in Cluster 2 and male-dominant in Cluster 4, further analysis revealed that this dimorphism was composed of lysophospholipids (LPLs), bile acids (BAs), and fatty acids **(**Fig. 4E-H**)**. The female-dominant Cluster 2 was characterized by higher counts of LPLs (females: 19, males: 6) and FAs (females: 12, males: 7). The male-dominant Cluster 4 had higher counts of LPLs (males: 30, females: 16), FAs (males: 17, females: 10), and BAs (males: 17, females: 9). Notably, a distinct female-specific advantage was also identified for FAs in Cluster 3, where the count in females was 2.3-fold greater than that in males (14 vs. 6, Fig. 4**G**). Structurally, this female advantage was even more pronounced for long-chain species (twofold) and saturated species (threefold) **(**Fig. 4J, M**)**. Detailed structural analysis of LPLs revealed that in Cluster 2, a female advantage was observed for long-chain and saturated species **(**Fig. 4K, N**)**. Conversely, in Cluster 4, a male advantage was evident for long-chain, very-long-chain, monounsaturated, and polyunsaturated LPL species **(**Fig. 4K, N**)**. In contrast, acylcarnitines and steroids were evenly distributed between sexes across all clusters, and the structural profiles of the acylcarnitines were also similar **(**Fig. 4E-H, I, L**)**.

Metabolic networks constructed from Pearson correlations revealed sex-specific architectural features (Figure S2A, B). The core modules in Clusters 1–3 were largely conserved, whereas those in Cluster 4 exhibited marked sex differences. Male networks were organized around BAs as central hubs (Figure S2A), whereas female networks were centered on highly unsaturated LPLs (Figure S2B). The female networks presented greater node density (84 vs 70) and connectivity (562 vs 467 connections).

### Sexually antagonistic TAG responses to acute exercise

Targeted TAG analysis revealed 172 TAG species across all the samples. Post-exercise alterations were observed in 77 male species and 66 female species **(**Fig. 5A-B**)**. Two-way repeated-measures ANOVA identified 37 TAG species with significant sex-by-exercise interaction effects (*P* < 0.05, Fig. 5C). Temporal heatmap analysis of the 37 interaction-significant TAGs revealed opposing regulatory patterns between the sexes **(**Fig. 5D**).** Structural characterization revealed distinct molecular features **(**Fig. 5E-H**)**. Analysis revealed that 94.6% of the samples contained even-numbered carbon chains, with C52 species accounting for 37.8% of all dimorphic TAGs **(**Fig. 5F**)**. The degree of unsaturation ranged from 1–6 double bonds, predominantly 2–5 bonds (86.5%) **(**Fig. 5G**)**. The most prevalent fatty acid components were palmitoleic acid (16:1, 29.7%) and oleic acid (18:1, 18.9%) **(**Fig. 5H**)**. The temporal dynamics of the five most statistically significant TAGs demonstrated consistent sex-specific patterns. The strongest sex-by-time interaction effects were observed for five TAG species, including TAG52:4(18:1), TAG50:2(16:1), and TAG50:3(16:1) (all -log₁₀*P* > 6.0, Fig. 5I**)**.

### Sex-dimorphicassociation between the exercise-induced metabolitesand* V̇*O_2peak_

Correlation analysis between the maximum absolute post-exercise metabolite perturbations (peak log_2_FC values) and *V̇*O_2peak_ revealed 115 metabolites with significant correlations in at least one sex. Among these, 48 metabolites (41.7%) exhibited opposite correlation directions between sexes **(**Fig. 6A**)**. In males, the most significant correlates included fatty acid metabolism-related metabolites (2,5-furandicarboxylic acid, dodecanedioic acid), organic acids (cresol-sulfate), and amino acid derivatives (methionine sulfone) **(**Fig. 6B**)**. In females, the most significant correlates were dominated by steroid sulfates (androsterone sulfate, epiandrosterone sulfate) and tryptophan catabolites (L-kynurenine, 5-hydroxyindoleacetic acid) **(**Fig. 6C**)**. Analysis of the five metabolites with the greatest sex differences (*ΔR*) revealed distinct patterns, including L-kynurenine, methionine sulfone, androsterone sulfate, 2,5-furandicarboxylic acid, and epiandrosterone sulfate (all *ΔR* > 0.65, Fig. 6D-H**)**.

## Discussion

This study presents the first comprehensive molecular atlas of sex differences in serum metabolic responses to acute exhaustive exercise in healthy young adults. Previous studies have established that during submaximal exercise, females demonstrate enhanced fat oxidation while males exhibit greater carbohydrate utilization, with these differences attributed to sex hormone profiles, body composition variations, and enzymatic activities [26–28]. However, the comprehensive molecular landscape underlying these substrate utilization differences under true physiological limits has remained largely unexplored [5, 29]. Under standardized termination criteria, objective data (RER_peak_, HR_peak_, and RPE) confirmed equivalent exhaustion states between sexes, and males and females still demonstrated profoundly distinct metabolic signatures **(**Table 1**).** As expected in this experimental design, males required greater absolute energy expenditure to reach these endpoints, reflecting established physiological differences in aerobic capacity and exercise tolerance [30, 31]. This study successfully implemented a rigorously controlled, standardized exhaustive exercise model, revealing that distinct sex-specific metabolic signatures exist even at equivalent levels of exhaustion. This comprehensive analytical approach provides a robust and reliable methodological template for future investigations into sex differences in exercise metabolism.

Exercise-induced metabolic responses are largely conserved between sexes, with 144 metabolites commonly upregulated and 137 commonly downregulated, representing 72–81% of all significantly altered metabolites **(**Fig. 2E, F**)**. AUC analysis revealed that the most prominent exercise-induced metabolites were common between sexes **(**Fig. 3D, E**)**, including established exercise biomarkers such as L-lactic acid, pyruvate, TCA cycle intermediates (citrate, isocitrate), L-acetylcarnitine, 3-hydroxybutyrate, Lac-phe, and N-acetylvaline, confirming the reproducibility of our data [6, 10, 20, 32]. However, distinct sex-dimorphic patterns emerged in sex-specific responses **(**Fig. 2**)**. Lipid species comprised the largest fraction of these sex-specific responses, accounting for 26–45% of sex-differentially abundant metabolites **(**Fig. 2E, F**)**, which is consistent with previous findings from submaximal exercise research that lipid metabolism represents a key metabolic signature distinguishing male and female exercise responses [33, 34]. These findings provide molecular evidence that exercise metabolism exhibits both conserved and sex-dimorphic response patterns.

Six metabolites emerged as the most consistent sex-discriminating markers across our analytical framework: hypoxanthine, sarcosine, methionine sulfoxide, lysophosphatidylcholine (LysoPC) species (sn1-LysoPC(16:1), sn2-LysoPC(16:1)), and 3-[3-sulfooxy]phenyl]propanoic acid **(**Fig. 3A-F**, **Figure S1A**)**. Hypoxanthine was increased in males, whereas sarcosine and 3-[3-sulfooxy]phenyl]propanoic acid were increased in females but decreased in males. Methionine sulfoxide and LysoPCs demonstrated greater consumption in males.

Hypoxanthine, the terminal product of ATP catabolism [35, 36], represents a direct indicator of total ATP turnover. Greater serum increases in males were consistent with our observation of longer exercise durations and greater total energy expenditure to reach standardized exhaustion endpoints, which aligns with previous submaximal exercise studies showing males’ greater reliance on carbohydrate utilization and the established role of hypoxanthine as an indicator of ATP turnover [37]. This pattern aligns with reported sex differences in purine metabolism. During exercise, males are known to exhibit reduced xanthine oxidase activity and increased efficiency of the purine salvage pathway [37, 38]. These established mechanisms provide strong support for the sex-dimorphic hypoxanthine responses observed in our study. LysoPC, a major subclass of LPLs generated by phospholipase A2-mediated phosphatidylcholine hydrolysis [39], is strongly correlated with skeletal muscle levels and force-generating capacity [40]. This relationship provides a mechanistic context for the observed sex-specific consumption patterns. Sarcosine and methionine sulfoxide, both one-carbon metabolism intermediates [41], exhibited opposite sex-specific patterns. While baseline sex differences in methylenetetrahydrofolate reductase and methionine sulfoxide reductase activities have been established in resting conditions [42], our findings extend these observations to demonstrate exercise-induced sex-dimorphic regulation of one-carbon metabolism pathways. Similarly, 3-[3-sulfooxy]phenyl]propanoic acid presented previously unreported sex-specific response patterns. These findings warrant systematic investigation of sex-related metabolic regulation during exhaustive exercise.

Temporal analysis revealed that fatty acids, LPLs, and BAs presented distinct sex-specific recovery patterns **(**Fig. 4**)**. Females showed sustained downregulation of these lipid classes throughout the 30-min recovery period (Cluster 2), whereas males demonstrated transient downregulation followed by restoration toward baseline levels (Cluster 4) **(**Fig. 4B, D**)**. These contrasting patterns may reflect fundamental differences in recovery metabolism between sexes. Sustained lipid downregulation in females is consistent with prolonged lipid oxidation during recovery, which aligns with established female preference for lipid metabolism from submaximal exercise studies [1, 43]. In contrast, the rapid restoration in males suggests a quicker transition in substrate utilization during the recovery phase. While the coordinated involvement of LPLs and BAs indicates broader metabolic regulation beyond simple fuel utilization, the functional significance of these changes in circulation requires further investigation. Analysis of the underlying network architecture revealed that while most metabolic connections were similar between sexes, certain clusters presented distinct organizational features (Figure S2A, B). In the most dimorphic cluster (Cluster 4), male networks were organized around BAs as hub metabolites, whereas female networks were more densely connected and centered on LPLs (Figure S2A, B). These findings suggest that sex differences in metabolic recovery may involve reorganization of specific network modules rather than holistic changes in metabolic architecture.

While multiple lipid classes showed substantial sex differences, TAGs were not covered by the untargeted platform. Since TAGs represent the primary storage form of fatty acids and are central to lipid metabolism [44], understanding their sex-specific exercise responses is of particular interest. Our targeted TAG analysis revealed compelling evidence of opposing regulation between sexes **(**Fig. 5**)**. A specific subset of TAGs, primarily C50‒C52 species rich in monounsaturated fatty acids, were consistently downregulated in males but upregulated in females post-exercise **(**Fig. 5A**‒**D**)**. This represents one of the clearest examples of sexually dimorphic metabolic regulation in our dataset. These findings extend previous observations from submaximal exercise studies showing greater post-exercise increases in circulating triglycerides in females than in males [45]. Our study demonstrated that this sex-dimorphic TAG response persists even under exhaustive exercise conditions and provides unprecedented molecular detail, revealing that this sex difference involves specific TAG species characterized by C50-C52 carbon chains, monounsaturated fatty acids, and palmitoleic acid-containing species.

Palmitoleic acid (16:1) was the most abundant monounsaturated fatty acid in these dimorphic TAGs **(**Fig. 5H**).** This aligns with our identification of sn1-LysoPC(16:1) as a major sex discriminator (Figure S1A). The consistent prominence of 16:1-containing lipids across platforms suggests that palmitoleic acid metabolism may be a key regulatory node in sex-specific exercise responses. Stearoyl-CoA desaturase 1 (SCD1), the rate-limiting enzyme that converts palmitic acid (16:0) to palmitoleic acid (16:1), likely mediates this coordinated regulation [46, 47]. Notably, free palmitoleic acid and palmitic acid pools showed no significant exercise-induced changes in either sex, suggesting that sex differences primarily involve 16:1 redistribution within complex lipids rather than alterations in free fatty acid pools. This pattern suggests possible metabolic coordination of 16:1 metabolism across lipid classes, although the specific regulatory mechanisms remain to be determined. However, our static concentration data cannot distinguish between synthesis, mobilization, and clearance differences, highlighting the need for future mechanistic studies.

Correlations between the magnitude of post-exercise metabolic perturbations and *V̇*O_2peak_ showed opposing patterns between sexes for a substantial proportion of metabolites **(**Fig. 6A**)**. L-kynurenine demonstrated striking sex-dimorphic fitness associations: positive correlations in males but negative correlations in females **(**Fig. 6D**)**. The kynurenine pathway represents the major tryptophan catabolism route activated by exercise through muscle PGC-1α1, which upregulates kynurenine aminotransferase (KAT), which converts kynurenine to neuroprotective kynurenic acid [48]. Higher fitness should theoretically be associated with more efficient kynurenine clearance, resulting in lower circulating levels. The opposing correlations suggest fundamental sex differences in pathway regulation; in females, greater kynurenine perturbations may indicate metabolic inefficiency, whereas in males, they may reflect greater metabolic flux capacity in different hormonal environments [49]. This sex-dimorphic pattern warrants investigation of downstream metabolites (kynurenic acid, quinolinic acid) to elucidate their functional significance.

Certain steroid hormone metabolites showed sex-specific associations with cardiorespiratory fitness. Androsterone sulfate, an androgen metabolite [50, 51], correlated positively with *V̇*O_2peak_ exclusively in females (Fig. 6F, H), suggesting that women with higher post-exercise levels of this metabolite exhibit greater cardiorespiratory fitness. Furthermore, betaine, a recently reported exercise-induced antiaging factor [53, 54], showed positive correlations with *V̇*O_2peak_ only in males (Fig. 6B), while its precursor proline betaine showed female-specific associations (Fig. 6C). These complementary patterns demonstrate that metabolites within related pathways can serve as sex-specific fitness biomarkers, emphasizing the necessity of sex-stratified approaches in exercise metabolism assessment. Certain steroid hormone metabolites showed sex-specific associations with cardiorespiratory fitness. Androsterone sulfate, an androgen metabolite [50, 51], correlated positively with *V̇*O_2peak_ exclusively in females **(**Fig. 6F, H**)**. This pattern may be related to the fact that in females, androgens are primarily of adrenal origin and exist within a lower baseline androgen environment compared to males [52]. Furthermore, betaine, a recently reported exercise-induced antiaging factor [53, 54], showed positive correlations with *V̇*O_2peak_ only in males **(**Fig. 6B**)**, while its precursor proline betaine showed female-specific associations **(**Fig. 6C**)**. These sex-specific patterns demonstrate that identical metabolites can exhibit distinct fitness associations between sexes, emphasizing the necessity of sex-stratified approaches in exercise metabolism research.

Several limitations should be acknowledged. The cross-sectional design precludes causal inference—observed metabolic differences represent correlative evidence, leaving unclear whether these differences drive, result from, or merely accompany sex-dimorphic physiological processes. Our findings are limited to healthy young adults aged 18–35 years, and the 30-min observation window may miss delayed sex-specific responses. Additionally, serum metabolite changes may not fully capture tissue-specific processes, and population-level findings may obscure substantial individual variation within each sex. Future longitudinal studies with tissue-specific analyses and individual-level characterization are needed to address these limitations.

Together, these findings provide unprecedented molecular insights into sex-specific exercise metabolism at the limits of human performance. The consistent patterns observed across multiple analytical approaches strengthen confidence in the biological significance of these sex differences and establish a foundation for future mechanistic investigations in precision exercise medicine.

## Conclusions

This study reveals distinct metabolic sexual dimorphism when males and females achieve standardized physiological exhaustion endpoints. Key findings include the identification of discriminating metabolites, sexually antagonistic TAG regulation, and opposing metabolic-fitness correlations exemplified by L-kynurenine and androsterone sulfate. The consistent prominence of palmitoleic acid-containing lipids suggests coordinated lipid metabolism regulation. These findings indicate that identical physiological endpoints correspond with distinct metabolic patterns between sexes, suggesting that sex-stratified approaches may enhance exercise biomarker interpretation and metabolic assessment protocols.

## Supplementary Information

Additional file 1.

## Data Availability

The datasets generated during the current study are not publicly available due to privacy restrictions but are available from the corresponding author upon reasonable request and with appropriate ethical approval.

## Abbreviations

ACSM: American college of sports medicine
ANOVA: Analysis of variance
ATP: Adenosine triphosphate
AUC: Area under the curve
BA: Bile acid
BMI: Body mass index
ChiCTR: Chinese clinical trial registry
CPET: Cardiopulmonary exercise testing
CV: Coefficient of variation
DXA: Dual-energy X-ray absorptiometry
ESI: Electrospray ionization
FA: Fatty acid
FDR: False discovery rate
FFM: Fat-free mass
HILIC: Hydrophilic interaction liquid chromatography
HMDB: Human metabolome database
HPLC: High-performance liquid chromatography
HR: Heart rate
IPAQ: International physical activity questionnaire
KAT: Kynurenine aminotransferase
LC-MS: Liquid chromatography-mass spectrometry
log₂FC: Log₂ fold change
LPL: Lysophospholipid
LysoPC: Lysophosphatidylcholine
MRM: Multiple reaction monitoring
MS/MS: Tandem mass spectrometry
m/z: Mass-to-charge ratio
OPLS-DA: Orthogonal partial least squares discriminant analysis
PAR-Q +: Physical activity readiness questionnaire plus
PCA: Principal component analysis
PGC-1α1: Peroxisome proliferator-activated receptor gamma coactivator 1-alpha 1
QC: Quality control
RER: Respiratory exchange ratio
RPE: Rating of perceived exertion
RPLC: Reversed-phase liquid chromatography
RSD: Relative standard deviation
SCD1: Stearoyl-CoA desaturase 1
sn1: Stereospecific numbering position 1
sn2: Stereospecific numbering position 2
TAG: Triacylglycerol
TCA: Tricarboxylic acid
TOF: Time of flight
UPLC: Ultra-performance liquid chromatography
*V̇*CO_2_: Carbon dioxide production
VIP: Variable importance in projection
*V̇*O_2_: Oxygen uptake
v/v: Volume per volume ratio
WHR: Waist-to-hip ratio

## Code availability

No custom code was developed for this study. All analyses were performed via standard statistical software and packages as described in the Methods section. The R scripts used for statistical analysis and data visualization are available from the corresponding author upon reasonable request.
