# Supplementary material for: Sexual dimorphism in the serum metabolome following acute exhaustive exercise
Source: Biol Sex Differ. 2025 Nov 6;16:91. doi: 10.1186/s13293-025-00780-x (PMC12593952; doi:10.1186/s13293-025-00780-x)
Supplement: Supplementary file 1 — Additional file 1. [file 13293_2025_780_MOESM1_ESM.pdf]

Supplementary Information

Supplementary Figure 1

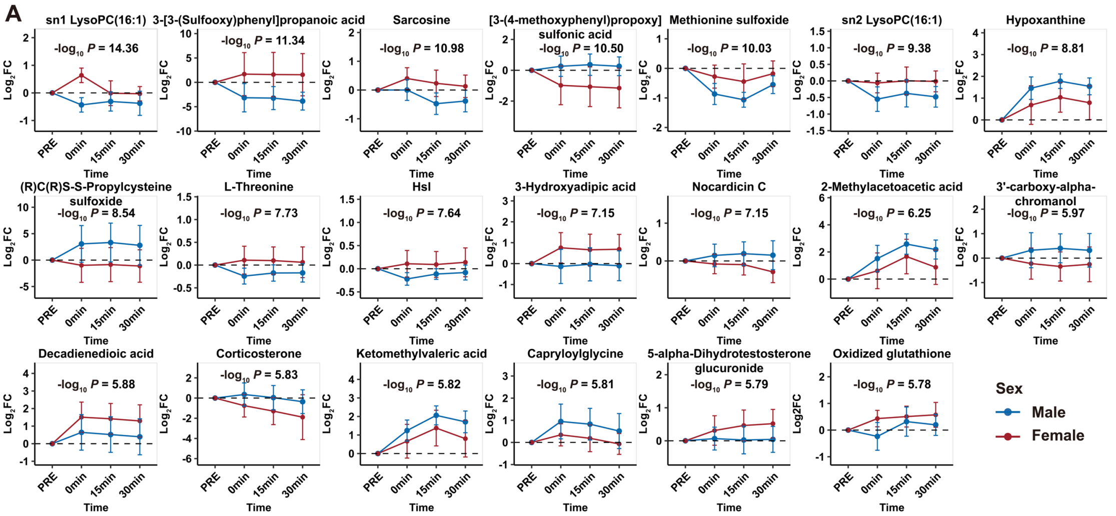

Figure S1 (A) Temporal dynamics of the top 20 metabolites ranked by sex main effect adjusted *P* values via two-way ANOVA; the blue lines represent males, the red lines represent females, the displayed -log10 adjusted *P* corresponds to the sex main effect, and the data show the means ± SDs.

Supplementary Figure 2

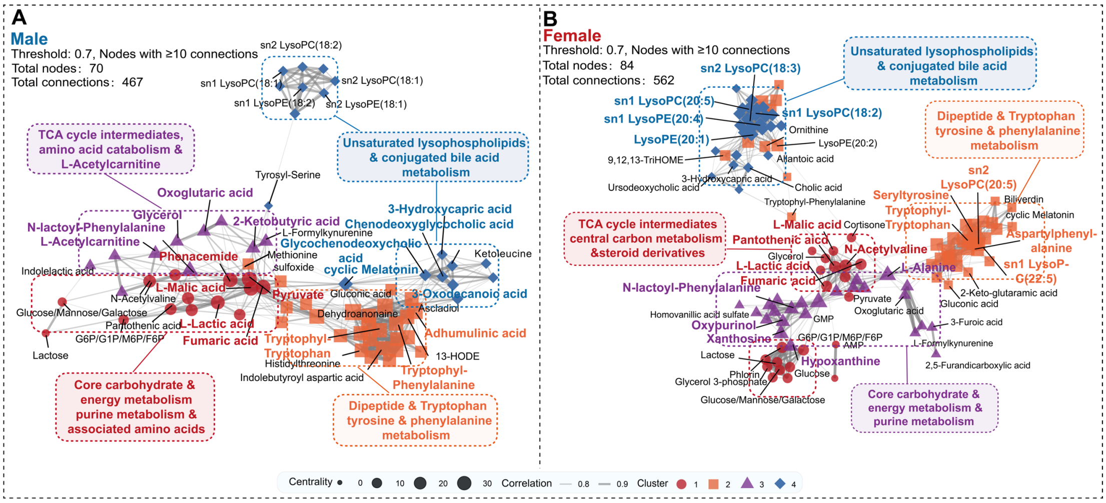

Figure S2 (A–B) Sex-specific metabolic networks based on Pearson correlations; threshold $|r| \geq 0.7$, $P < 0.05$, displaying nodes with ≥10 connections, node shapes and colors indicate temporal cluster membership.
